# Kinase and Phosphatase Engagement Is Dissociated Between Memory Formation and Extinction

**DOI:** 10.3389/fnmol.2019.00038

**Published:** 2019-02-20

**Authors:** Mario Rafael Pagani, Emiliano Merlo

**Affiliations:** ^1^Instituto de Fisiología y Biofísica Bernardo Houssay (IFIBIO)-Houssay, Facultad de Medicina, Universidad de Buenos Aires—Consejo Nacional de Investigaciones Científicas y Técnicas (CONICET), Buenos Aires, Argentina; ^2^Department of Psychology, University of Cambridge, Cambridge, United Kingdom

**Keywords:** associative memory, acquisition, consolidation, extinction, kinase, phosphatase

## Abstract

Associative long-term memories (LTMs) support long-lasting behavioral changes resulting from sensory experiences. Retrieval of a stable LTM by means of a large number of conditioned stimulus (CS) alone presentations produces inhibition of the original memory through extinction. Currently, there are two opposing hypotheses to account for the neural mechanisms supporting extinction. The unlearning hypothesis posits that extinction affects the original memory trace by reverting the synaptic changes supporting LTM. On the contrary, the new learning hypothesis proposes that extinction is simply the formation of a new associative memory that inhibits the expression of the original one. We propose that detailed analysis of extinction-associated molecular mechanisms could help distinguish between these hypotheses. Here we will review experimental evidence regarding the role of protein kinases and phosphatases (K&P) on LTM formation and extinction. Even though K&P regulate both memory processes, their participation appears to be dissociated. LTM formation recruits kinases, but is constrained by phosphatases. Memory extinction presents a more diverse molecular landscape, requiring phosphatases and some kinases, but also being constrained by kinase activity. Based on the available evidence, we propose a new theoretical model for memory extinction: a neuronal segregation of K&P supports a combination of time-dependent reversible inhibition of the original memory [CS-unconditioned stimulus (US)], with establishment of a new associative memory trace (CS-noUS).

## Introduction

Having a brain allows animals to integrate environmental and internal sensory information to generate adaptive behaviors. In some cases, these behaviors are acquired through an interaction of actual experiences and the animal’s intrinsic behavioral repertoire. Our current understanding of these behavioral phenomena posits that while learning is a behavioral change as a function of somatosensory experience, memory is the maintenance of that change for a given period of time. Whereas forming adaptive memories is beneficial, maximizing fitness in ever changing environmental conditions, sometimes it is necessary to inhibit existing outdated memories through a process called memory extinction. Memory formation and extinction are multi-level psychobiological phenomena that require changes in neuronal connectivity supported by intricate neural and molecular events. In humans, persistent maladaptive memories are at the core of psychiatric disorders such as anxiety disorders (e.g., post-traumatic stress disorder and specific phobias) or drug addiction (Everitt and Robbins, 2016; Walsh et al., 2018). Understanding better the molecular underpinnings that support memory formation and extinction would be key to design and implement more effective clinical interventions to treat these psychiatric conditions.

## Formation and Extinction of Associative Memories. Behavioral and Mechanistic Insights

Learning can lead to the formation of short- or long-term memories (STM, LTM) by altering neuronal connectivity within specific brain networks. Associative memory formation depends on the contingent occurrence of a previously irrelevant environmental stimulus (conditioned stimulus, CS) with a biologically relevant environmental or internal stimulus (unconditioned stimulus, US). USs that may reduce survival probabilities (e.g., presence of a predator or sickness) result in aversive memories, whereas USs that may enhance survival probabilities (e.g., food or mate) result in appetitive memories. As a consequence of successful associative learning the behavioral response typically associated with the US becomes a conditioned response (CR) under CS control.

The capacity to form associative memories is an evolutionary conserved feature, present in most species within the Animal Kingdom. For example, adult *Drosophila* flies trained with pairings of an electric shock (US) with an odor (CS) will avoid the CS during subsequent memory tests, evidencing the formation of an associative CS-US LTM (Tully and Quinn, 1985). Similarly, rodents trained with contingent presentations of an auditory cue (CS) and an electric footshock (US) will freeze to later presentations of the CS alone, a typical defensive behavior normally elicited by the US (Blanchard et al., 1975).

Research conducted in diverse animal paradigms has revealed conserved fundamental mechanisms supporting associative memory formation. CS-US memory traces are sensitive to disruption during or shortly after learning and require a period of consolidation in order to become stable and long-lasting. LTM consolidation is a time-dependent process entailing *de novo* mRNA and protein synthesis (Katche et al., 2013). Memory consolidation recruits specific neuronal subpopulations within distinctive brain regions, with the basolateral amygdala (BLA) and the dorsal hippocampus acting as key hubs within an extended network (Han et al., 2007; Grewe et al., 2017).

**Figure 1:**
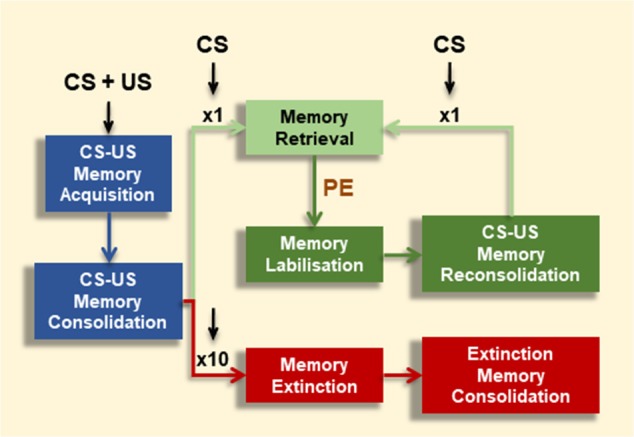
Effect of conditioned stimulus (CS) alone presentations on associative memory fate. Contingent CS-unconditioned stimulus (US) presentations promote memory formation (Memory Acquisition), which might consolidate into long-term memory (LTM) over time under appropriate training conditions (Memory Consolidation). CS-exposure could have alternative effects depending on number or extent of CS events. A brief CS presentation triggers the conditioned response (CR; Memory Retrieval). However, under certain boundary conditions (e.g., prediction error signal, PE), this brief CS presentation leads to memory destabilization (Memory Labilization), from which the memory becomes stable again by a restabilization process (Memory Reconsolidation). After reconsolidation, the original memory persists, shown by the maintenance of the CR at subsequent retrieval (Memory Retrieval). Alternatively, after repeated CS presentations (e.g., 10 CSs) the CR is inhibited (Memory Extinction).

Once fully consolidated, associative memories can undergo alternative retrieval-dependent memory processes (Figure 1). Depending on the extent of CS-alone exposure, CS-US memories can be maintained or inhibited (Merlo et al., 2014). A brief CS presentation session can result in memory destabilization followed by a (so-called) reconsolidation process. Reconsolidation leaves the CR intact, and is proposed as an updating opportunity in face of new information regarding the CS-US association (Pedreira et al., 2004; Lee, 2008). Even though consolidation and reconsolidation of an associative memory share the requirement of mRNA and protein synthesis (Nader et al., 2000; Duvarci et al., 2008), they are dissociable at the molecular level (Lee et al., 2004; Lee and Hynds, 2013) On the contrary, extensive CS-alone exposure results in memory extinction and inhibition of the CR. Memory extinction was first described by Pavlov and Anrep (1927) and is an evolutionary conserved mechanism (Pedreira and Maldonado, 2003; Merlo et al., 2014; Felsenberg et al., 2017; Hermann et al., 2017; Nishijima and Maruyama, 2017). Memory extinction is characterized by distinctive features (i.e., spontaneous recovery, renewal, reinstatement and savings) which indicate that extinction does not erase the original CS-US memory trace, but transiently inhibits it by the formation of a new associative memory (CS-noUS) as a consequence of CS-alone trials (Todd et al., 2014). Successful memory extinction is defined by effective short- or long-lasting CR reduction towards the CS after repeated CS-alone presentations, rather than the mere experimental manipulation. As for CS-US memories, long-term extinction requires a period of consolidation where *de novo* mRNA and protein synthesis are required (Vianna et al., 2003; Santini et al., 2004).

To date, there are two alternative hypotheses that account for extinction. On the one hand, the new learning hypothesis posits that extinction entails the formation of a new inhibitory memory trace formed by the association of the CS and the absence of the US (new learning hypothesis; Todd et al., 2014; Felsenberg et al., 2018). On the other hand, extinction may be underpinned by an unlearning mechanism partially affecting the original memory trace, hence reducing or preventing it from taking behavioral control upon later CS presentations (unlearning hypothesis; Mauk and Ohyama, 2004; Delamater and Westbrook, 2014; Khalaf et al., 2018). Experimental observations at both neural and molecular levels indicate that both alternative processes could be required to explain extinction mechanisms. Within the BLA, an extinguished CS specifically activates “extinction neurons” and fails to activate “fear neurons” which were active after fear conditioning (Herry et al., 2008). Moreover, extinction reverts structural modifications induced by the formation of the original memory (Lai et al., 2012) presumably by counteracting memory formation at the subcellular level (Schwaerzel et al., 2002). Whereas Herry and colleagues’ results could be explained by either the alternative extinction hypotheses, Lai et al. (2012) results clearly show that extinction *affects* the CS-US ensemble neurons in a way that opposes the structural changes caused by memory formation.

At the molecular level, memory formation and extinction rely on both common and exclusive mechanisms. For instance, whereas both processes depend on N-methyl-D-aspartate-type glutamate receptor (NMDAR) activity (Lee and Kim, 1998; Wu et al., 2007; Zimmerman and Maren, 2010), they require activation or inhibition of NF-κB- (Merlo et al., 2005; Merlo and Romano, 2008; de la Fuente et al., 2011) or AP-1-dependent gene expression (Guedea et al., 2011), respectively. Hence, the molecular events linking NMDAR activation with NF-κB- or AP-1-dependent gene expression under consolidation of the original memory or its extinction should be fundamentally different. One could argue that if memory extinction was solely supported by the formation of an inhibitory CS-noUS memory trace, it would rely on qualitatively similar molecular mechanisms to formation of the original memory. We, and others (Delamater and Westbrook, 2014; Clem and Schiller, 2016), believe that a combination of these two alternative (and apparently opposing) hypotheses provides the best theoretical framework to account for most of the behavioral, pharmacological and molecular empirical data to date.

Fear conditioning in rodents is the most used behavioral paradigm to study neuronal and molecular mechanisms in associative memory mechanisms. However, given their easily accessible and relatively simple central nervous systems, invertebrates such as the sea snail *Aplysia*, fruit flies, crabs and honey bees, had contributed significantly to this research area. Among the complex molecular events subserving memory formation and extinction, we will review the role of protein kinases and phosphatases (K&P). These two protein families are vital for the integration of chemical extracellular information (e.g., glutamate release) and intracellular states that lead to specific gene expression regulation (e.g., recruitment of constitutive or inducible transcription factors), two well established events supporting such behavioral changes. Despite fundamental neuroanatomical differences between vertebrates and invertebrates, empirical data support an evolutionary conserved role of K&P for memory formation and extinction. Here, we will show that K&P together define the course and quality of memory formation and extinction in a specific and evolutionarily conserved manner. Moreover, we will propose a new theoretical model to account for the molecular mechanisms subserving memory extinction.

Due to the widespread use of fear conditioning in rodents, most of the scientific evidence reviewed here has been obtained in that behavioral paradigm. When available, we will include additional key evidence supporting and expanding specific topics coming from other learning paradigms.

## Synaptic Plasticity Subserving Memory Formation and Inhibition

During cued fear conditioning, a sub-population of lateral amygdala (LA) pyramidal neurons react to the US (an electric footshock) with a strong depolarization, which contributes to strengthen the response to the contingent CS (a tone; Grewe et al., 2017). Thus, in a Hebbian manner, a weak neuronal activation produced by the tone is strengthened by the temporal pairing with the strong input produced by the US (Hebb, 1949; LeDoux, 2000). The necessary synaptic plasticity underlying fear conditioning is proposed to be supported by long-term potentiation (LTP) mechanisms. For decades, LTP has been proposed as the cellular basis of memory formation and many correlations with memory mechanisms have been described (Martin et al., 2000). However, a causal relationship between LTP induction and memory formation has been shown just recently. Nabavi et al. (2014) showed that fear conditioning can be induced in animals by pairing an electric footshock with optogenetic stimulation of a light-activated channel ChR2 of LA auditory inputs. This artificial optogenetic training had the ability to implant a fear memory since treated animals tested by the presentation of a tone alone displayed a CR. Remarkably, the artificially implanted memory was inactivated by optogenetic low frequency stimulation that produced long-term depression (LTD), as well as reactivated by optogenetic high frequency stimulation that produced LTP. These experiments showed that synaptic strengthening by LTP in LA permits the retrieval of a memory, whereas synaptic weakening by LTD prevented retrieval. Of note, the optogenetic stimulation alone, without the presentation of the electric shock did not produce a CR to the test tone. Similarly, unpaired presentation of the US and the optogenetic-CS produced neither LTP nor a CR. These results, among a considerable number of previous reports, strongly associate synaptic plasticity in the form of LTP and LTD with memory formation and inhibition. Moreover, expression and inhibition of a fully consolidated fear memory are linked to potentiation and depotentiation of the same set of synaptic contacts. These data add support to the interpretation that extinction of a fully consolidated associative memory specifically affects synaptic contacts within the CS-US memory ensemble.

## Memory Acquisition

CS-US memory formation can be divided into processes of memory acquisition and memory consolidation. Memory acquisition refers to neural and molecular events that influence behavioral performance during or shortly after training. On the contrary, memory consolidation refers to neural and molecular processes involved in the establishment of a long-lasting memory.

### K&P Regulation of Glutamate Receptors

K&P regulate a number of key molecular partners supporting memory acquisition, including NMDA-type and α-amino-3-hydroxy-5-methyl-4-isoxazolepropionic acid-type (AMPA) glutamate receptors, and Ca^2+^/calmodulin-dependent protein kinase II (CaMKII). Postsynaptic NMDARs in LA are essential for establishment of LTP and associative memory. As coincidence detector of pre- and postsynaptic contingent activity NMDARs are permeable to Ca^2+^, which in turn promotes autophosphorylation of CaMKII Thr286 residue (Giese et al., 1998). CaMKII autophosphorylation promotes prolonged kinase activation by switching from a Ca^2+^-dependent to a Ca^2+^-independent mode (Miller and Kennedy, 1986). Whereas CaMKII acting on a number of molecular substrates contributes to different aspects of fear memory, its action on GluA1 subunits of AMPARs contribute specifically to AMPAR insertion in the postsynaptic membrane (Hayashi et al., 2000), a major event in LTP and memory acquisition (Malinow and Malenka, 2002). Consistently, pharmacological blockade of NMDARs and CaMKII in the amygdala has been shown to prevent memory acquisition (Fanselow and Kim, 1994; Rodrigues et al., 2004).

Protein kinase A (PKA) and C (PKC), as well as CaMKII, phosphorylate NR1, NR2A, and NR2B NMDAR subunits in different sites (Omkumar et al., 1996; Leonard and Hell, 1997; Tingley et al., 1997). However, the role of these post-translational modifications in associative learning is unclear. The role of PKA as a regulator of NMDAR activity and LTP has been reported consistently, with PKA activity enhancing NMDAR total currents and Ca^2+^ influx in cell cultures and hippocampal slices (Skeberdis et al., 2006). Similarly, protein tyrosine kinases enhance NMDAR-dependent currents and Ca^2+^ influx (Wang and Salter, 1994). Intra-BLA injection of PKA inhibitor H7 blocks contextual and cued fear memory acquisition (Goosens et al., 2000).

NMDAR activation in neurons also produces activation of protein phosphatases 1 (PP1). In particular, PP1 is activated by Ca^2+^ influx through synaptic NMDARs (Hou et al., 2013). PP1 activity regulates LTD and LTP induction in opposite ways, with PP1 being required for LTD but opposing LTP induction *via* CaMKII dephosphorylation (Jouvenceau and Dutar, 2006). In hippocampal slices cAMP-dependent PP1 inhibitor 1 (PP1-I1) activation results in increased CaMKII Thr286 phosphorylation, leading to a Ca^2+^-independent CaMKII sustained activity (Blitzer et al., 1998). Consistently, genetic inhibition of PP1 within the cortex and hippocampal formation facilitates two forms of associative learning, novel object recognition and spatial navigation (Genoux et al., 2002). Even though these two types of memories are associated with specific concerted actions of hippocampal LTD and LTP (Wang et al., 2004; Goh and Manahan-Vaughan, 2013), they are both equally influenced by PP1 inhibition. Similar to PP1, the PP1 calcineurin (CaN; also known as PP2B) is activated by Ca^2+^ influx through NMDARs and reduces the opening probability of the receptor in a typical negative feedback loop mechanism (Lieberman and Mody, 1994). Reduction of hippocampal CaN levels by intraventricular-administration of CaN antisense oligodeoxynucleotide facilitates *in vivo* hippocampal LTP and enhances contextual fear memory (Ikegami et al., 1996; Ikegami and Inokuchi, 2000). Transgenic expression of a CaN specific inhibitor driven by CaMKIIα promoter in the brain reduces CaN activity in the hippocampus and cortex, facilitates hippocampal LTP *in vitro* and improves short- and long-term object recognition memory and LTM of spatial navigation (Malleret et al., 2001).

### Neuromodulatory Inputs Regulating K&Ps

The firing of a third neuromodulatory category of neuron, other than the pre- and postsynaptic neurons described above, also contributes to synaptic plasticity and memory acquisition. Norepinephrine (NE) and dopamine (DA) have been implicated in fear conditioning. Both neurotransmitters, binding to G-protein-coupled receptors, regulate adenylate cyclase with the subsequent modulation of intra-cellular cAMP levels (Cerne et al., 1993; Raman et al., 1996). NE and DA release increases in LA after US presentation (Yokoyama et al., 2005) and receptors for both neurotransmitters are expressed in LA (Muly et al., 2009; Farb et al., 2010). Beta-adrenergic receptor (β-AR) activation promotes PKA-mediated enhancement of NMDAR-dependent excitatory post-synaptic currents in the hippocampus. Such an effect appears to be constitutively downregulated by CaN (Raman et al., 1996). Moreover, in medium spiny neurons of the nucleus accumbens DA-dependent PKA activation also phosphorylates the NR1 NMDAR subunit, which is sensitive to PP1/PP2A dephosphorylation (Snyder et al., 1998). This evidence indicates that PP1 and CaN constraining effects on memory formation could be due, at least in part, to alteration of DA or NE signaling recruited by training.

Typically, if a drug reduces STM and LTM when it is administered *before* training, but has no effect when administered after training, it is interpreted that it affects learning or memory acquisition. However, if the drug reduces LTM when is administered *after* training, it is interpreted that it affects memory consolidation. This reasoning may have some caveats, as discussed presently. Pre-training administration of propranolol, a non-selective antagonist of β-ARs, produced amnesia for auditory fear conditioning. Systemic or intra-LA application of propranolol after training or before memory retrieval had no effect on memory, suggesting that β-ARs were not involved in either memory consolidation or retrieval (Cole and Koob, 1988; Bush et al., 2010). However, later studies showed that during training β-ARs initiate two different molecular processes, AMPARs phosphorylation at Ser845, which contributes to memory acquisition, and extracellular regulated kinase (ERK) activation, which contributes to memory consolidation (Schiff et al., 2017). Thus, when an intervention affects LTM, leaving STM intact, it should be considered as affecting memory consolidation, regardless of the manipulation time relative to training.

Numerous reports have implicated DA signaling on various forms of associative learning, including aversive and appetitive conditioning. The requirement of DA neuromodulation for associative memory formation and extinction appears to be a conserved property in vertebrates and invertebrates (Burke et al., 2012; Abraham et al., 2016). In general DA has a positive modulatory effect on memory processes, but the specific effect on K&Ps in the postsynaptic partner depends on the extent to which D1-like or D2-like receptors are engaged. The complexity of DA signaling with respect to brain regions, receptor activation and effect on memory process far exceeds the objective of this review (for a specific review see Abraham et al., 2014).

Together, these findings show that protein kinases acting directly or indirectly on glutamate receptors enable synaptic events leading to synaptic plasticity and memory formation. On the contrary, PP1s appear to counteract these processes, obstructing memory formation. DA and NE neuromodulatory inputs modulate memory by affecting the balance between K&P.

## Memory Consolidation

Immediately after learning the newly acquired CR is sensitive to disruption by a number of interventions, including amnestic drugs. However, after several hours, the recently established memory becomes stabilized and insensitive to amnestic interventions. This process of memory stabilization is commonly known as cellular consolidation or simply consolidation. In contrast to memory acquisition, a large number of molecular mechanisms have been identified for consolidation. Here we review the most relevant data to discuss the role of (K&P) in memory consolidation.

Memory consolidation involves the activation of constitutive and inducible transcription factors, which regulate gene expression to support changes in synaptic strength and learning in key brain regions (Alberini and Kandel, 2014). A conceptual framework, on which we build our discussion, can be summarized as follows: after training, activated kinases (e.g., PKA, MAPK) activate transcription factors (e.g., NF-κB, CREB or AP-1), which leads to gene transcription and protein translation that provides effector proteins for structural synaptic modifications. These molecular landscapes underlying associative memory consolidation were originally observed in *Aplysia*’s gill and siphon withdrawal reflex mediated by long-term sensitization (Kandel, 2001), but then confirmed and expanded in other animals. Memory consolidation mechanisms constitute an evolutionarily conserved feature of associative LTM formation present from *C. elegans* to mammals.

### sCaMK, PKA and MAPK

In cued and contextual fear conditioning, intra-BLA administration of PKA, MAPK, or protein synthesis inhibitors immediately after training (Rp-cAMPS, PD098059 and anisomycin, respectively) disrupt LTM while leaving STM intact (Goosens et al., 2000; Schafe and LeDoux, 2000; Schafe et al., 2000). CaMKII is also required for memory consolidation. Intra-hippocampal or amygdala injection of the CaMKII inhibitor KN62 administered immediately after inhibitory avoidance training disrupted LTM (Wolfman et al., 1994). Similarly, CaMKII transgenic inhibition in the forebrain impaired LTM formation in contextual and cued fear conditioning (Mayford et al., 1996). Another Ca^2+^-calmodulin dependent kinase, CaMKIV, is required for contextual fear LTM formation without affecting STM (Wei et al., 2002). Kinase activity of CaMKII, CaMKIV, PKA and the MAPK ERK1/2 contributes to CREB-dependent gene expression in neurons (Gonzalez and Montminy, 1989; Dash et al., 1991; Bito et al., 1996; Bartsch et al., 1998; Impey et al., 1998; Kang et al., 2001). In addition, in contextual fear conditioning Ca^2+^/calmodulin-dependent adenylate cyclase signaling is required for the sequential activation of ERK, the CREB kinase MSK1 and finally CREB. Interestingly, activation of ERK and CREB co-localizes with PKA into the same hippocampal CA1 neuronal subpopulation (Sindreu et al., 2007). In line with the proposed role of these kinases in consolidation, ERK1/2 is transiently activated by associative learning experience in the LA and its blockade specifically affects LTM, but not STM (Schafe et al., 2000). Similar results have been observed in mice and invertebrates, including *Aplysia*, *Drosophila* and the crab *Neohelice granulathus* (Shalin et al., 2004; Feld et al., 2005; Fioravante et al., 2006; Pagani et al., 2009). Activation of CaMKs, PKA, ERK1/2 and MSK1 leads to the phosphorylation and activation of the transcription factor CREB, which in turn conduct the cAMP response element (CRE)-dependent gene expression necessary for memory consolidation (Lisman et al., 2018). As a proof of concept, disruption of CRE-dependent gene expression by means of a αCaMKII promoter-dependent inducible expression of a CREB repressor fusion protein disrupts LTM formation, without altering STM (Kida et al., 2002). This pattern of results, with intact STM and disrupted LTM, are characteristic of manipulations that alter memory consolidation specifically, and has been a gold standard for discovering consolidation mechanisms for the last three decades.

Although CREB has been at the center of studies on memory consolidation mechanisms, its role as a gene expression regulator is not unique, but in concert with the action of other transcription factors. For instance, NMDAR-dependent hippocampal ERK1/2 activation induced by an inhibitory avoidance training activates Elk-1 (Cammarota et al., 2000). Also in an ERK-dependent manner, induction of AP-1-dependent gene expression in the hippocampus mediates contextual fear memory consolidation (Guedea et al., 2011). Consolidation of fear memories in crabs, rats and mice requires the activation of the constitutive transcription factor NF-κB. Pharmacological blockade of Iκ-B kinase (IKK), responsible for NF-κB activation, or silencing of κB-dependent gene expression by specific oligonucleotides results in intact STM, but impaired LTM, for aversive associative memory in mice, rats and crabs (Romano et al., 2006).

So far, we have reviewed experimental data linking kinase activity with memory formation in the context of transcriptional activation. Next, we will review the role of other, less studied, kinases in memory formation. As mentioned above, ERK1/2 is a major molecular component of memory consolidation, though other MAPKs, including ERK5, c-JNK1–3, and p38 are also involved in memory formation (Adams and Sweatt, 2002). The kinase p38 is activated in the dorsal hippocampus after inhibitory avoidance training, and its inhibition impairs both STM and LTM (Alonso et al., 2003). c-JNK function in memory is still under debate. Intra-hippocampal administration of c-JNK inhibitor SP600125 enhanced LTM of contextual fear conditioning (Sherrin et al., 2010) but blocked inhibitory avoidance memory (Bevilaqua et al., 2003). Bevilaqua and colleagues also observed a memory enhancing effect of SP600125 but only affecting STM. The observation of c-JNK activity as a memory constraining mechanism is the only report so far of kinase activity opposing the establishment of an associative memory (but also see section “Cdk5 in plasticity and memory”). Clearly, further studies are needed to solve this discrepancy and establish the true nature of c-JNK involvement during memory formation.

### PKA and CaMKII Functions Are Restrained by PP1, CaN and STEP

In contrast to the requirement of CaMKII and PKA, PP1 appears to restrain LTM consolidation since transgenic inhibition of PP1 facilitates spatial and novel object recognition LTMs. PP1 inhibition enhances CREB and CaMKII activity in cortex and hippocampus, suggesting that the phosphatase constrains canonical memory mechanisms (Genoux et al., 2002). Also, PP1 appears to be a major regulator of histone phosphorylation in the nucleus (Koshibu et al., 2009), another mechanism required for LTM formation through an MSK1-dependent histone phosphorylation (Chwang et al., 2007). Similarly, the PP1 Inhibitor-2 (PP1-I2) also controls CREB activation and CREB-dependent gene transcription in fear conditioning. Lentiviral knockdown of PP1-I2 in the dorsal hippocampus does not affect STM, but enhances LTM, possibly through positive regulation of PP1 and suppression of CREB-mediated gene expression (Yang et al., 2015).

The phosphatases CaN and striatal-enriched protein tyrosine phosphatase (STEP) are both activated by exposing rats to a novel context and have comparable effects on LTD and LTP (Yang et al., 2006). Pharmacological inhibition of CaN or STEP in CA1 hippocampal neurons promotes synaptic potentiation (Wang and Kelly, 1996, 1997; Pelkey et al., 2002). Conversely, overexpression of CaN or STEP impairs long-lasting hippocampal LTP (Winder et al., 1998; Paul et al., 2007). CaN effects on synaptic plasticity are mediated, at least in part, by dephosphorylation of AMPAR GluR1 subunit at Ser845. This post-translational modification of AMPARs regulates receptor trafficking, open-state probability and membrane insertion (Banke et al., 2000; Beattie et al., 2000; Man et al., 2007). Interestingly, PKA activation increases Ser845 phosphorylation (Price et al., 1999).

In relation to memory mechanisms, CaN was also reported as a key component of memory consolidation, but in an opposite way to PKA or MAPK. Overexpression of a truncated CaN in the hippocampus prevented LTM, but left STM intact, in spatial navigation and object recognition (Mansuy et al., 1998). Similarly, CaN inhibition by anti-sense oligodeoxynucleotides or transgenic interventions in dorsal hippocampus enhanced associative memory formation (Ikegami and Inokuchi, 2000; Malleret et al., 2001). As with CaN, STEP knockout (KO) mice show improvement in hippocampal-dependent memory tasks. This effect may be mediated by an increase in ERK1/2 activation, and NMDA and AMPA receptor phosphorylation in the hippocampus (Venkitaramani et al., 2011). On the contrary, intra-BLA administration of a substrate-trapping STEP, a hyperactivated version of the phosphatase, prevents activated ERK1/2 nuclear translocation and disrupts fear memory consolidation (Paul et al., 2007).

### Paradoxical Relationship Between PI3K and PTEN Actions on Memory Formation

Another important phosphorylation signaling pathway for CREB-dependent gene transcription involves phosphatidylinositol-3-kinase (PI3K) and AKT. Activated PI3K phosphorylates phosphatidylinositol (4,5)-bisphosphate, producing phosphatidylinositol (3,4,5)-triphosphate (PIP3) in the plasma membrane. PI3P serves as a substrate and molecular dock for many signaling mechanisms including AKT and PKC (Vanhaesebroeck et al., 2010). PI3K activation within the BLA is mediated by two distinct mechanisms: activation of tyrosine kinase receptors (e.g., TrkB), and Ca^2+^ influx through NMDARs (Lin et al., 2001; Ou and Gean, 2006). Notably, brain-derived neurotropic factor (BDNF) induction of TrkB promotes ERK1/2 activation by either activation of Ras/Raf/MEK signaling pathway, or activation of PI3K (Lin et al., 2001, 2003b; Ou and Gean, 2006). These BDNF-dependent signaling mechanisms appear necessary to promote fear LTM in lateral and basal amygdala, but not in hippocampus or cortex. Similarly, PI3K-dependent signaling within the BLA is necessary for LTM (Lin et al., 2001; Ou and Gean, 2006). Additionally, AKT regulates mTOR, a kinase required for translation control in synaptic plasticity and fear memory consolidation (Hoeffer and Klann, 2010).

The phosphatase and tensin homolog (PTEN) counteracts PI3K function. Inducible deletion of PTEN in adult mice impairs hippocampal LTD and LTP, spatial memory (Wang et al., 2006; Sperow et al., 2012) and contextual fear conditioning (Lugo et al., 2013). Interestingly, deletion of PDK1 (3-phosphoinositide-dependent protein kinase 1) rescues the defect in synaptic plasticity but not spatial memory observed in PTEN mutant mice (Sperow et al., 2012). Even though PTEN counteracts PI3K, they are both necessary for plasticity and memory consolidation. This paradox may be explained by recent evidence indicating that PTENα dephosphorylates CaMKII at T305/306 and T337. This molecular event makes CaMKII activatable by promoting its interaction with NR2B subunits of NMDARs (Wang et al., 2017). Consistently, mutant mice lacking PTENα activity show a deficit in LTP as well as in fear conditioning and spatial memory (Wang et al., 2017).

### Cdk5 in Plasticity and Memory

The cyclin-dependent kinase 5 (Cdk5) is involved in cytoskeletal dynamics, affecting synaptic transmission and plasticity (Lalioti et al., 2010). The Ca^2+^-dependent protease calpain can cleave p35, a regulatory subunit of Cdk5, producing p25, which in turn promotes Cdk5 activity. p25 fragments are generated in the hippocampus after spatial learning (Engmann et al., 2011). Transient overexpression of p25 in hippocampus enhances cued and contextual fear and spatial memory (Angelo et al., 2003; Fischer et al., 2005). Moreover, pharmacological or genetic inhibition of Cdk5 in lateral septum or hippocampus in mice impairs contextual fear and spatial memory (Fischer et al., 2002, 2003; Guan et al., 2011), suggesting p25/Cdk5 pathway is necessary for memory formation. Nevertheless, long-term Cdk5 loss enhanced synaptic plasticity and spatial memory, probably by reducing NR2B NMDAR subunit degradation (Hawasli et al., 2007). Hence, Cdk5 affects synaptic activity and memory formation in opposite ways depending on the timescale of the manipulation (Fischer et al., 2005). Importantly, acute pharmacological manipulations are consistent and indicate that under physiological conditions Cdk5 is required for associative LTM formation.

### Shp2 a Phosphatase Promoting Plasticity and Memory

Most phosphatases involved in learning and memory appear to inhibit plasticity and memory formation. However, the Src homology 2-containing tyrosine phosphatase (Shp2) appears to promote LTM formation in olfactory conditioning in *Drosophila* (Pagani et al., 2009), and contextual fear conditioning and spatial navigation in mice (Kusakari et al., 2015; Zhang et al., 2016; Yan et al., 2017). Although Shp2 is not required for STM, during training it contributes to ERK1/2 activation and is critical for the trial spacing effect affecting LTM formation (Pagani et al., 2009). In terms of Shp2 function, it has been shown to be required for the BDNF-induced complete activation of RAS/ERK (Easton et al., 2006), and contributes to LTP induction by regulating AMPARs trafficking as well as NMDARs phosphorylation (Zhang et al., 2016; Yan et al., 2017).

### Role of Fyn, LIMK and PTPRR on Memory Formation

Even though there is little information about the role of these enzymes on memory mechanisms, the available data is enough to make meaningful comparisons between memory formation and extinction processes.

Contextual fear conditioning activates the tyrosine kinase Fyn, a member of the Src kinase family, in mouse dorsal hippocampus. Fyn-deficient mice show no increase in tyrosine phosphorylation after fear conditioning in the hippocampus, and also fail to form STM and LTM (Isosaka et al., 2008) suggesting that Fyn is required for memory formation.

LIM kinase 1 and 2 (LIMK1/2) are involved in cytoskeleton dynamics and synaptic plasticity. LIMK KO mice showed altered excitatory synaptic plasticity, with an enhanced hippocampal LTP induction in LIMK1/2 or LIMK1 KO compared to wild type and LIMK2 KO mice (Meng et al., 2002, 2004). LIMK1 KO mice showed mild auditory fear conditioning enhancement, but also severe morphological alterations in synaptic structures (Meng et al., 2002). Pharmacological inhibition of LIMK by intra-hippocampal BMS-5 administration immediately after training disrupts contextual fear memory, but not memory extinction (Lunardi et al., 2018), indicating an exclusive participation of LIMK on memory formation.

Another phosphatase involved in memory mechanisms is the protein tyrosine phosphatase receptor type R (PTPRR). Homozygous KO mice for PTPRR show enhanced fear conditioning compared to wild types, but normal fear memory extinction and spatial learning. Importantly, elevated freezing behavior after training could not be due to a generalized elevated anxiety in the KO mice since their displayed similar levels of anxiety to wild types in the mirrored chamber test (Erkens et al., 2014). This pattern of results indicates that PTPRR acts as a constraint during memory formation, but does not take part in memory extinction.

## Memory Extinction

In comparison to memory formation mechanisms, memory extinction mechanisms are known in much less detail. Here, we review experimental evidence addressing the participation of K&P in extinction. As with CS-US memory formation, these studies are diverse in terms of the animal species, types of memory and experimental approaches used.

### Protein Phosphatases

The involvement of CaN (also known as PP2B) in memory extinction has been studied in auditory and contextual fear memory, the fear-potentiated startle response and conditioned taste aversion in rats and mice.

Lin et al. (2003a, b) showed that extinction of fear-potentiated startle response in rats increases BLA CaN expression. Administration of the CaN inhibitors FK506 or cypermethrin intra-BLA, or cyclosporin A intravenously, disrupted extinction of the fear-potentiated startle response. These data were the first indication that, contrary to the role of CaN constraining CS-US memory acquisition, the phosphatase is necessary for extinction memory formation.

Extinction of auditory fear conditioning in rats also increases BLA CaN protein levels in the cytosolic fraction. Reduction of CaN expression by means of intra-BLA injection of an oligodeoxynucleotide against the A and B subunits of CaN does not affect extinction learning but does disrupts extinction consolidation. Interestingly, analysis at the single animal level shows a negative correlation between fear reduction and CaN expression, suggesting that not only is CaN required for memory extinction, but also that its BLA expression level indicates extinction intensity (Merlo et al., 2014). Extinction of contextual fear memory is also dependent on CaN activity. This was shown by interfering with CaN activation state either by intra-hippocampal injection of FK506 (de la Fuente et al., 2011) or transgenic inhibition of CaN in the forebrain (Havekes et al., 2008). Intriguingly, forebrain transgenic CaN inhibition or activation failed to affect extinction of conditioned taste aversion in mice (Baumgärtel et al., 2008) suggesting that extinction of some associative memory types is CaN-independent.

As mentioned above, the role of PP1 during memory formation is to constrain the process. Unfortunately, there is virtually no information regarding the role of PP1 in memory extinction. In an *in vitro* extinction model in *Hermissenda crassicornis*, PP1 inhibition prevents decreases in B-cell spike frequency, suggesting that PP1 is a key component in extinction mechanisms (Cavallo et al., 2014). Investigating the role of PP1 in behavioral extinction is necessary and will help to better understand the mechanisms of memory inhibition through extinction.

In contextual fear conditioning in rodents, intra-hippocampal NSC87877, an inhibitor of Shp2, disrupts memory extinction but does not affect fear memory formation (Isosaka and Yuasa, 2010). The discrepancy between this study and the ones showing a requirement of Shp2 on memory formation (see section “Shp2 a phosphatase promoting plasticity and memory”) could be explained by procedural differences, species or pharmacokinetics of Shp2 inhibitor NSC87877 compared to transgenic manipulations of Shp2 gene. But also, given the role of Shp2 in mediating the effect of spacing learning trials, it is probable that under the training conditions used by Isosaka and Yuasa, Shp2 was not recruited for memory formation. Altogether, these results suggest that Shp2 activity is a necessary mechanism for the formation and extinction of associative memories.

In relation to the other phosphatases studied on CS-US memory formation and mentioned in the previous section, PTPRR, STEP and PTEN, their role on memory extinction remains poorly understood. The only one that has been experimentally evaluated during memory extinction is PTPRR, which has been shown to not be required (Erkens et al., 2014). More research is needed to understand the contribution of STEP and PTEN to this memory process.

### Kinases Necessary for Memory Extinction

#### ERK1/2

ERK1/2 is a key molecule for memory extinction mechanisms. Intra-BLA or dorsal hippocampus administration of ERK1/2-dependent intracellular pathway inhibitors U0126 or PD9859 impair auditory and contextual fear memory extinction (Herry et al., 2006; Fischer et al., 2007). Moreover, extinction enhancement by the NMDAR partial agonist D-cycloserine is mediated by an increase on BLA ERK1/2 activation (Merlo et al., 2018). Besides being a common mechanism between memory formation and extinction, ERK1/2 activation shows specific time scales for each process. Whereas ERK1/2 is activated in the BLA 1 h after fear conditioning, after extinction training it is activated within 20 min (Schafe et al., 2000; Merlo et al., 2014). Even though ERK pharmacological inhibition blocks both memory acquisition and extinction, some findings suggest fundamental differences in its role during each process. For instance, the requirement for ERK-dependent signaling for memory acquisition and extinction in the hippocampus is associated with opposing regulation of cFos and GluR2/3 gene expression (Guedea et al., 2011).

#### p38

Another member of the MAPK family, p38 is required for memory extinction. Blockade of p38 activity, by intra-hippocampal administration of the specific inhibitor SB203580 immediately after extinction sessions, prevented the extinction of inhibitory avoidance memory. The fact that the amnestic effect on extinction was present when the injection was given immediately after but not 180 min later, and that the inactive analog of SB203580 (SB202474) was ineffective, strongly indicates that the extinction impairment is specific to p38 blockade (Rossato et al., 2006).

#### CASK

A kinase with apparent specific participation in memory extinction is the Ca^2+^/calmodulin-dependent serine kinase (CASK). The role of CASK on memory processes has been tested by using transgenic mice carrying a point mutation in the protein, T470A. T470 is a PKA phosphorylation site, and when phosphorylated allows CASK to increase the transcriptional activity of T-Brain-1, an immediate early gene associated with synaptic plasticity (Chuang et al., 2014). CASK T470A mice show normal auditory fear conditioning and conditioned taste aversion, but impaired extinction of both memories. Notably, T470A mice show normal acquisition and extinction of contextual fear memory (Huang and Hsueh, 2017).

#### c-JNK

Micro-injections of the c-JNK specific inhibitor SP600125 into the dorsal hippocampus immediately after daily extinction sessions prevented inhibitory avoidance extinction in comparison with vehicle treatment (Bevilaqua et al., 2007). Moreover, CA1 neurons within the dorsal hippocampus show an increase in c-JNK/phosphoERK interaction induced by extinction of contextual fear memory. Through this interaction, memory extinction results in a specific increase in phosphorylation of c-JNK in Ser63/73 and Ser243, in contrast to a reduction of p-c-JNK Ser243 observed after acquisition. These findings suggest that fear memory extinction requires a concerted action of ERK and c-JNK reducing the expression of AP-1-dependent genes (Guedea et al., 2011).

#### α-Ca^2+^/CaMKII (αCaMKII)

αCAMKII T286A point mutation prevents the autophosphorylation activity of the kinase, weakening the binding of calmodulin and shortening its activation state (Rodrigues et al., 2004). Whereas homozygous αCaMKII^T286A−/−^ mice have defects on contextual fear memory acquisition, heterozygous αCaMKII^T286A+/–^ mice can learn the task and have been used to analyze the participation of this kinase on memory extinction. Heterozygous αCaMKII^T286A+/–^ mice show impairments in both contextual fear and spatial memory extinction tasks (Kimura et al., 2008). The differential sensitivity of memory acquisition and extinction to homo- or heterozygosity suggests a differential involvement of αCaMKII, with extinction showing more sensitivity to alterations in αCaMKII activity. Furthermore, pharmacological inhibition of αCaMKII in dorsal hippocampus by local injection of the inhibitor KN-62 prevents extinction of inhibitory avoidance memory (Szapiro et al., 2003).

#### LIM Kinase (LIMK)

LIMKs are serine protein kinases that regulate actin and microtubule reorganizations in various cell types (Bernard, 2007). Intra-hippocampal injection of LIMK inhibitor BMS-5 20 min before extinction training had no effect on extinction or spontaneous recovery of contextual fear memory. Interestingly, the same pharmacological intervention impaired fear memory acquisition (Lunardi et al., 2018), suggesting LIMK is a specific for the establishment of the original memory but not for extinction.

#### PI3K

Using the PI3K inhibitor LY294002 administered into the dorsal hippocampus immediately after extinction training, Chen et al. (2005) showed that the kinase is necessary for memory extinction. At the synaptic plasticity level, hippocampal slices of mice with disrupted PI3Kγ subunit or slices from wild type mice treated with the selective PI3Kγ inhibitor AS-605240 showed NMDAR-dependent LTD deficits. Unexpectedly, PI3Kγ deficient mice and mice injected with AS-605240 intra-hippocampally did not show defects in contextual fear memory consolidation or extinction (Kim et al., 2011), suggesting that other isoforms of PI3K could explain the results obtained with LY294002.

## Kinases Constraining Memory Extinction

In contrast to its role during memory acquisition, IKK activity constrains memory extinction. In crabs, systemic administration of the specific IKK inhibitor sulfasalazine before extinction training delays spontaneous recovery of the original memory (Merlo and Romano, 2008). Moreover, extinction training induces NF-κB inhibition in crab brain, confirming that an extinction-mediated IKK inactivation is required for the establishment of an extinction memory. In mice, contextual fear memory extinction also requires a functional reduction in NF-κB activity in dorsal hippocampus (de la Fuente et al., 2011), supporting an evolutionarily conserved function of IKK inactivation during memory extinction. Even though pharmacological blockade of IKK resulting in enhanced extinction could be interpreted as an experimental artifact, the fact that NF-κB is inhibited in the brain by extinction training suggests that IKK inactivation is an intrinsic extinction mechanism.

Extinction training inhibits another memory-related kinase, Cdk5. Intra-hippocampal injection of Cdk5 inhibitor butyrolactone I enhances contextual fear extinction. Consistently, time-specific over-expression of the Cdk5 activator p25 results in increased Cdk5 activity in dorsal hippocampus, and when p25 expression is induced before extinction training prevents contextual fear extinction (Sananbenesi et al., 2007). As with IKK, memory extinction inhibits Cdk5.

Another kinase necessary for memory formation, Fyn, has opposite effect on extinction. Contextual fear extinction specifically downregulates Fyn activity in the dorsal hippocampus. Pharmacological inhibition of Fyn by intra-hippocampal injection of PP2 facilitated fear extinction (Isosaka et al., 2009).

The role of PKA on extinction has been shown to be either a requirement or a constraint, depending on the brain region and type of memory. In inhibitory avoidance, intra-hippocampal administration of the PKA inhibitor Rp-cAMPs prevents extinction (Szapiro et al., 2003), indicating hippocampal PKA activity is necessary for extinction of this memory. On the contrary, extinction of contextual fear conditioning is constrained by PKA activity. Increasing PKA activity in the forebrain by means of post-natal transgenic overexpression of adenylate cyclase type-1 delayed extinction (Wang et al., 2004), while decreasing its activity in hippocampus, amygdala, temporal and prefrontal cortex by overexpression of a PKA dominant negative form R(AB) enhanced extinction (Isiegas et al., 2006). Similarly, disrupting intra-cellular PKA compartmentalization in dorsal hippocampus by micro-injection of membrane permeable PKA anchoring disrupting peptides St-Ht31 or St-superAKAP-IS enhanced contextual fear extinction (Nijholt et al., 2008). Hence, PKA is necessary for acquisition and extinction of inhibitory avoidance, but produces opposing effects in acquisition and extinction of contextual fear conditioning.

Altogether, these data indicate that PKA, IKK, Cdk5 and Fyn have opposing roles on the acquisition or extinction of purely Pavlovian memories, such as contextual fear conditioning.

## Molecular Landscapes Associated with Memory Formation and Extinction

The experimental evidence presented so far aims to establish modes of kinase and phosphatases actions on memory formation and extinction in order to better understand the nature of these memory mechanisms. This information has been gathered in different species and behavioral paradigms, and constitute a prime example of independent and convergent empirical evidence. This type of knowledge, far from weakening our understanding of memory mechanisms, brings strong support to conserved and general principles of such complex phenomenon. The intention of this review is not to generate a complete list of molecular events associated with these memory processes, but to analyze representative examples where the enzymatic activities have been tested both in memory formation and extinction. Exceptions to this rule are to highlight areas were more research is needed. In Table 1 we present a summary of the literature presented in previous sections.

**Table 1:** Role of kinases and phosphatases (K&P) on associative memory formation and extinction.

		Acquisition/Consolidation	Extinction
**Kinases**	ERK	Necessary	Necessary
	CASK	Not involved	Necessary
	Fyn	Necessary	Constrain
	c-JUN	Constrain/necessary	Necessary
	Cdk5	Necessary	Constrain
	PKA	Necessary	Constrain
	p38	Necessary	Necessary
	LIMK	Necessary	Not involved
	IKK	Necessary	Constrain
	αCamKII	Necessary	Necessary
	PI3K	Necessary	Necessary
**Phosphatases**	Calcineurin	Constrain	Necessary
	PP1	Constrain	Necessary?
	PTPRR	Constrain	Not involved
	STEP	Constrain	?
	PTEN	Necessary	?
	Shp2	Necessary	Necessary

The first clear conclusion from Table 1 is that memory formation requires PKActivation in different brain regions. This is a well-documented feature of both STM and LTM formation. Training for associative memory induces the activation of several kinases, and their activity is necessary in order to acquire and/or consolidate that learning experience and form a memory. These kinases have different activation requirements and modes of actions, but their activation would be important to support changes in synaptic connectivity by affecting existing proteins or promote synthesis of new ones through translation of existing or new mRNAs. Apparently, the only exception to this rule is c-JNK, for which contradictory evidence has been reported (Bevilaqua et al., 2003; Sherrin et al., 2010).

The next conclusion is that most of the phosphatases studied during memory induction restrain memory mechanisms. In other words, when CaN or PP1 are inhibited during training, the resulting associative memory is enhanced, which means it would be retained for longer or be more resistant to extinction. Phosphatase action during memory acquisition or consolidation would specifically counteract the phosphorylation of kinases activated by the learning experience. Removal of this counteracting action results in more intense or prolonged kinase activation and a larger impact of their positive effect in memory establishment. Altogether, this evidence indicates that associative memory consolidation results from a balanced and coordinated action of K&P in key neurons within specific brain regions. A balance favoring kinase activation will result in activation of constitutive and inducible transcription factors and mRNA and protein synthesis. On the contrary, when the balance favors phosphatase activity memory consolidation is compromised.

The only two examples of phosphatases being necessary for memory formation are PTEN and Shp2. Probably, this difference is due to the fact that Shp2 and PTEN promote excitatory synaptic potentiation in contrast to the canonical role of phosphatases constraining excitatory synaptic potentiation and promoting LTD (Baumgärtel and Mansuy, 2012; Connor and Wang, 2016).

Analysis of K&P involvement in memory extinction produces a more heterogeneous molecular landscape. Among the kinases discussed here, ERK, PI3K, CaMKII and p38 have similar effects when subserving memory formation or extinction, as they are all required. A similarly frequent set of kinases show a different profile, being necessary for memory formation but constraining memory extinction. This is the case for IKK, PKA, Fyn and Cdk5, whose inhibition during memory extinction facilitated extinction learning and consolidation. The latter group is particularly interesting from a theoretical perspective, since it strongly supports a fundamental difference between memory formation and extinction mechanisms (see below). The role of phosphatases during extinction has attracted much less attention, but the available data are indicative of such fundamental differences. The paradigmatic example of this disparity is the role of CaN, of which activity is required for memory extinction. Interestingly, CaN is associated to memory formation and inhibition in a similar way to synaptic plasticity. Whereas CaN constrains associative memory formation and LTP, it is required for memory extinction and LTD induction. Shp2 is also required for extinction, but in contrast to CaN it is also required for CS-US memory formation. Even though there are no reports on the role of PP1 or STEP in memory extinction, their involvement could be similar to CaN, as suggested for some preliminary *in vitro* analysis.

These molecular landscapes also present rare kinase and phosphatase examples. The kinases LIMK and CASK are specifically associated to memory formation or extinction, respectively; whereas the phosphatase PTPRR is specifically involved in establishment of the original memory. Suggesting there are a subset of K&P that are specific to one or the other memory mechanism is appealing, but more research needs to be done to understand fully the contribution of these partners on memory mechanisms.

## Extinction as A Combination of Memory Formation and Inhibition

At first sight, the increased molecular heterogeneity underpinning CR inhibition suggests that extinction mechanisms go beyond the formation of a new CS-noUS inhibitory associative memory trace. Moreover, extinction could be conceptualized as a combination of associative memory promotion mechanisms, in charge of CS-noUS memory formation, and memory depression events, associated with a temporary inhibition of the original CS-US trace.

**Figure 2:**
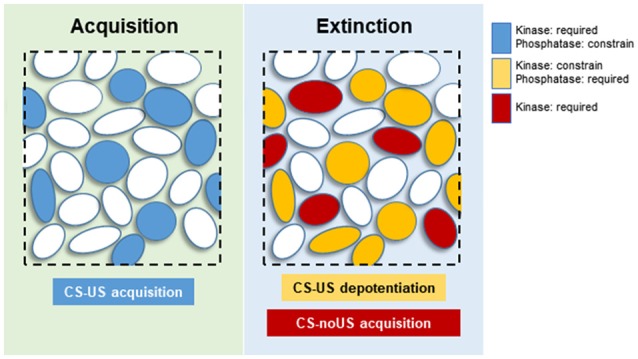
Proposed neuronal distribution of kinases and phosphatases (K&P) during memory acquisition and extinction. Blue circles: neurons participating in CS-US memory acquisition, requiring activation of kinases, and constrained by phosphatases. Yellow circles: “fear neurons” undergoing synaptic depotentiation, requiring the activity of phosphatases and constrained by kinase activities. Red circles: “extinction neurons” responsible for acquisition of the new CS-noUS memory through activation of various kinases.

At the neuronal level, CS-US and extinction memories are supported by distinctive neuronal subpopulations within specific brain regions. In the BLA, high CS fear is associated with activation of “fear neurons” characterized for their response to the CS in fear conditioned animals. Similarly, when these animals extinguish the fear memory, the “fear neurons” stop responding to the CS, but a new neuronal subpopulation is engaged and specifically respond to the extinguished CS. These so-called “extinction neurons” did not respond to the CS when the animals were in a high CS fear state (Herry et al., 2008). In the dentate gyrus (DG), activation of contextual fear memory recall-induced neurons is required for memory extinction, indicating that extinction affects the original memory trace at a cellular level (Khalaf et al., 2018). These results indicate that extinction affects both BLA and DG original CS-US memory trace neurons at an engram-specific level by silencing or changing the valence of neuronal activation. We propose that specific subsets of K&P are associated with “fear” and “extinction neurons.” During fear conditioning, the coordinated action of K&P will help in establishing a neuronal ensemble of “fear neurons” within the BLA, associated to other specific neuronal subpopulations in other key brain regions (e.g., mPFC or dorsal hippocampus). For this reason, the molecular landscape of CS-US memory formation would be more homogeneous, with kinases promoting, and phosphatases controlling, the sculpture of an internal CS representation that closely resembles the US representation (Figure 2; Grewe et al., 2017). Extinction would comprise a much more diverse set of events. Synaptic or excitability changes affecting the response of “fear neurons” to the CS would be under the control of PP1s such as CaN. Complementary synaptic changes would affect a new neuronal population, the “extinction neurons,” where activation of kinases, transcriptional and translational machinery would lead to the establishment of a new associative CS-noUS memory trace (Figure 2). Under this theoretical framework, those kinases that constrain memory extinction would be acting specifically on “fear neurons,” counteracting the effect of CaN or other required phosphatases.

This model of memory extinction allows for the development of testable hypotheses to understand better the underlying mechanisms. For instance, this model predicts the segregation of specific molecular mechanisms subserving extinction in specific brain regions. Using anatomical assessment and pharmacological manipulations it should be possible to test if the activation of K&P required for extinction are independent molecular events, taking place in different neuronal populations. Moreover, it should be interesting to analyze whether phosphatase activation affects specifically neurons allocated to the CS-US neuronal ensemble.

## Concluding Remarks

Here, we have reviewed the participation of K&P for the formation and extinction of associative memories. The available evidence strongly suggests that extinction mechanisms exceed, in complexity and variety, the mechanisms sub-serving the establishment of the original memory trace. We propose a novel theoretical frame-work explaining the complexity of memory extinction and generating testable new hypotheses. Memory extinction is the basis of current treatments for anxiety disorders such as post-traumatic stress disorder or specific phobias. Aversive conditioning in humans appears to have similar properties to those described in animal models (Martin-Soelch et al., 2007). Therefore, many extinction mechanisms presented here are expected to be relevant for humans as well. Hence, expanding and improving our understanding of memory inhibition mechanisms would be crucial to improve existing treatments or design new ones.

## Author Contributions

EM and MP designed and wrote the manuscript.

## Conflict of Interest Statement

The authors declare that the research was conducted in the absence of any commercial or financial relationships that could be construed as a potential conflict of interest.
